# The moderating role of internet addiction severity on the relationship between affective temperaments and emotion regulation in adolescents and young adults

**DOI:** 10.3389/fpsyg.2025.1682156

**Published:** 2025-09-23

**Authors:** Carmela Mento, Daniele Mollaioli, Chiara La Barbiera, Federica Arena, Francesca Capellupo, Maria Rosaria Anna Muscatello, Clara Lombardo

**Affiliations:** ^1^Department of Biomedical, Dental and Morphological and Functional Imaging Sciences, University of Messina, Messina, Italy; ^2^Department of Clinical and Experimental Medicine, University of Messina, Messina, Italy; ^3^Department of Health Sciences, University “Magna Graecia” of Catanzaro, Catanzaro, Italy; ^4^Psychiatric Unit, Polyclinic Hospital, Messina, Italy; ^5^University of Messina, Messina, Italy

**Keywords:** affective temperaments, cognitive style, emotional regulation, internet addiction, mental health

## Abstract

**Introduction:**

Adolescence and young adulthood are critical periods for the development of emotion regulation, a process increasingly influenced by the pervasive use of digital technologies. This study investigated whether the severity of Internet Addiction (IA) moderates the relationship between affective temperaments and emotion regulation strategies in adolescents and young adults with problematic Internet use.

**Method:**

Data were collected from a convenience sample of 262 Italian participants (62.6% female; aged 13–21) via an online survey between March and December 2024. Participants completed validated measures of Internet addiction (IAT), affective temperaments (TEMPS-A), and emotion regulation strategies (ERQ), and analyzed with different moderation models for each affective temperament.

**Results:**

Moderation analyses revealed that IA severity significantly weakened the negative association between depressive and anxious temperaments and cognitive reappraisal, and also reduced the positive link between anxious temperament and suppression, indicating that higher problematic Internet use may buffer some temperament-related emotion regulation difficulties.

**Discussion:**

Findings suggest that IA severity moderates the link between affective temperaments and emotion regulation. Specifically, higher IA levels weakened the negative association between depressive/anxious temperaments and cognitive reappraisal, and also reduced the positive link between anxious temperament and suppression, indicating that problematic Internet use may buffer some temperament-related emotion regulation difficulties. Future longitudinal research is needed to clarify the long-term impact of this digital scaffolding on emotional development.

## Introduction

Adolescence and young adulthood represent critical developmental phases where stable personality traits, such as affective temperaments, and the capacity for emotion regulation play a fundamental role in shaping psychological well-being and adaptation to life’s challenges (Balle et al., 2022; Fombouchet et al., 2023).

Affective temperaments, as conceptualized in Akiskal’s model, include five main dimensions: cyclothymic, depressive, irritable, hyperthymic, and anxious (Akiskal et al., 2005). These temperamental traits represent stable personality characteristics that influence emotional and behavioral patterns across different contexts. They are deeply implicated in the way individuals process and regulate emotions, which, in turn, impacts their ability to cope with stressors and challenges.

On the other hand, emotion regulation represents a critical psychological process that enables individuals to manage their emotional experiences effectively and adapt to daily challenges (Savci, 2017).

In this developmental context, technology has become a fundamental aspect of everyday life in recent years, profoundly shaping adolescence and early adulthood. Excessive use of the Internet, social media, and digital technologies can contribute to psychological distress and promote risky online behaviors, which are frequently linked to mental health issues (Bickham, 2021).

The so-called “new addictions” including technodependence, have become deeply rooted in modern society. Unlike substance-related addictions, these emerging forms of dependency were considered marginal until recent times (Pan et al., 2020). Recent evidence suggests that early and pervasive exposure to digital technologies is associated with both increased psychological distress and changes in social and emotional development, raising concerns about potential long-term consequences of excessive online engagement (Twenge and Campbell, 2018; Przybylski and Weinstein, 2019). Moreover, technology addiction among young people can be just as disabling, with effects comparable to those of substance addiction.

While the concept of technodependence encompasses a more diverse range of problematic behaviors, reflecting the increasing impact of technology on various aspects of daily life (Agarwal and Kar, 2015; Huang et al., 2020), several constructs have been proposed to describe specific problematic technology use, such as Internet Gaming Disorder (IGD) and Internet Addiction Disorder (IAD) (Ko, 2014; Kurniasanti et al., 2019).

The IGD specifically refers to compulsive and problematic online gaming behaviors that significantly impairs daily functioning, while IAD represents a specific form of addiction related to compulsive Internet use in a range of online activities beyond gaming – such as social networking, surfing and streaming – that leads to significant psychological, social, sexual, academic or work impairment (Block, 2008; Petry et al., 2015; Mollaioli et al., 2018).

Research has consistently highlighted that excessive Internet use has been linked to alterations in the prefrontal cortex that can impair executive functions, negatively impact daily life, and increase the risk of suicidal behaviors among adolescents (Block, 2008; Li et al., 2014; De Stefano et al., 2022). Digital technology and Internet may also act as a form of cognitive scaffolding, an external structure that alters the cognitive demands of a task by providing support to individuals with emotional regulation difficulties (Vygotsky, 1980).

In the context of IAD, specific affective temperaments may predispose individuals to adopt maladaptive emotion regulation strategies, exacerbating compulsive behaviors and psychological distress (Tsai et al., 2020). For instance, anxious temperaments are associated with heightened emotional reactivity and increased vulnerability to stress, which could amplify susceptibility to problematic Internet use (Akiskal et al., 2005). Similarly, cyclothymic and depressive temperaments, characterized by mood instability and negative affectivity, may hinder effective emotion regulation, further contributing to dependency on digital technologies as a coping mechanism.

Similarly, problematic use of Internet may emerge as maladaptive emotion regulation strategies in which the digital environment supports and amplifies emotional and cognitive processes that individuals have difficulty managing internally. This scaffolding might initially compensate for self-regulation deficits but, over time, foster over-reliance on technology to modulate emotions (Lavallee, 2023).

Liang et al. (2021) emphasized the mediating role of emotion regulation strategies – such as cognitive reappraisal and expressive suppression – in the relationship between negative emotional states and Internet addiction in adolescents. Their findings suggest that an inability to manage emotions effectively may exacerbate problematic Internet use, highlighting the importance of targeted interventions to improve emotion regulation skills. Similarly, other studies have demonstrated that difficulties in emotion regulation are closely linked to the development of dysfunctional behaviors, including IAD (Sheppes et al., 2015; Gioia et al., 2021).

Despite the growing body of research on Internet addiction, the specific interplay between affective temperaments and emotion regulation strategies remains insufficiently explored, particularly in younger populations. Given that stable personality traits are widely recognized as key factors in shaping emotion regulation processes (Gross, 2008; Ozturk et al., 2013; Purnamaningsih, 2017; Hughes et al., 2020), it is crucial to investigate whether and how affective temperaments contribute to these dynamics.

Understanding the role of affective temperaments in emotion regulation can provide valuable insights into individual differences in coping mechanisms. This study aims to fill this gap by examining these relationships through a comprehensive cross-sectional design. Considering this, the present study aims to examine whether the severity of Internet Addiction moderates the relationship between affective temperaments and emotion regulation in a sample of adolescents and young adults with problematic Internet use, using validated psychometric instruments. The findings could provide new perspectives for the development of targeted interventions aimed at promoting more adaptive emotion regulation and reducing vulnerability to technological addiction.

## Methods

### Study design and population

A cross-sectional study was conducted via an online survey between March and December 2024 with 306 Italian participants aged 13–21. This age range was specifically chosen as it encompasses late adolescence and young adulthood, a critical developmental period characterized by the maturation of emotion regulation skills and a heightened vulnerability to the onset of behavioral addictions, including problematic Internet use (Brook et al., 2025).

The selected socio-demographic data and psychological instruments were entered into a panel of online data collection tools (e.g., Google Forms^®^) and sent throughout Italy as an invitation to participate in the research, which consisted of filling out an online survey disseminated through social network web advertising, institutional or other professional mailing lists and messaging services.

All participants were provided with written information on the research objectives, methods and expected benefits. Data were appropriately anonymized and informed consent was obtained at the time of the original data collection; for the participation of minors in the study, consent was obtained from parents or guardians. Participants were excluded if they scored below 30 on the Internet Addiction Test (IAT), had less than 8 years of education, or were over the age of 30. Of the 306 eligible participants, 12 were excluded due to incomplete survey responses, and 32 were excluded for failing to meet the inclusion criteria. The final sample of 262 participants were identified in two subgroups: Mild IA (*n* = 149) and Moderate IA (*n* = 113) according to IAT score and related severity levels (24). The study was conducted with participants complying with the Declaration of Helsinki and was approved by the local ethics committee of Centre for Psychological Research and Intervention (Ce. R. I. P.) for studies involving humans (Prot, n. 0011424 del 31/01/2024).

### Measures

The following psychological tests were administered:

- The Internet Addiction Test (IAT), developed by Young (1998), is a 20-item questionnaire designed to assess various dimensions of Internet use. Each item is rated on a 5-point Likert scale, ranging from 0 (least extreme behavior) to 5 (most extreme behavior). The total score is calculated by summing the responses of all 20 items, resulting in a maximum possible score of 100. Higher scores indicate greater severity of compulsive Internet use. Total scores that range from 0 to 30 points are considered to reflect a normal level of Internet usage; scores of 31 to 49 indicate the presence of a mild level of Internet addiction; 50–79 reflect the presence of a moderate level; scores of 80–100 indicate a severe dependence upon the Internet. The test shows good internal consistency, with Cronbach’s alpha values reported between 0.83 and 0.86 (25, 26). The Italian adaptation of the IAT showed solid psychometric properties, including reliability and discriminant and convergent validity (Fioravanti and Casale, 2015).
- The Temperament Evaluation of Memphis, Pisa, Paris, and San Diego (TEMPS-A) (Akiskal et al., 2005) is a self-report questionnaire used to assess five temperamental dimensions in both psychiatric populations and healthy individuals. The short version of the TEMPS-A consists of 39 items derived from the original extended version of 110 items. It assesses the temperaments cyclothymic (items 1–12), depressive (items 13–20), irritable (items 21–28), hyperthymic (items 29–36) and anxious (items 37–39). Respondents answer each item dichotomously (“Yes” or “No”) and specific temperamental traits are identified according to cut-off scores: 8 or more “Yes” responses for depressed, 4 or more for cyclothymic, 6 or more for hyperthymic, 3 or more for irritable and 10 or more for anxious temperaments. The TEMPS-A has been translated into numerous languages, has been extensively validated and demonstrates good reliability of its subscales (Cronbach’s *α* > 0.70) (Preti et al., 2010).
- The Emotion Regulation Questionnaire (ERQ) (Balzarotti et al., 2010) is a 10-item self-report instrument designed to measure two emotion regulation strategies: cognitive reappraisal (6 items) and expressive suppression (4 items). Responses are rated on a 7-point Likert scale ranging from “strongly disagree” to “strongly agree.” The Italian version of the ERQ demonstrated good internal consistency, with reliability coefficients of 0.84 for cognitive reappraisal and 0.72 for expressive suppression, as well as acceptable two-month test–retest reliability (0.67 for reappraisal and 0.72 for suppression). These results are consistent with the psychometric properties of the original English version (Gross and John, 2003).

### Statistical analysis

All statistical analyses were conducted using R version 4.4.2 (R Core Team, 2025), using the *lme4*, *interactions*, *dplyr*, *broom*, and *knitr* packages. For all inferential statistical analyses, the significance level was set at *p* < 0.05. Descriptive statistics were calculated to summarize the demographic characteristics of the study sample and the distribution of key study variables, including affective temperaments and emotion regulation strategies. Means, standard deviations, and percentages were computed where appropriate.

Prior to conducting inferential analyses, normality of the study variables was assessed using skewness and kurtosis (acceptable range: ±1.0) and visually inspected through Q–Q plots. Additionally, the assumption of homoscedasticity was evaluated for independent sample t-tests through Levene’s Test to confirm equal variances between groups.

Group comparisons were performed to assess differences in affective temperaments and emotion regulation strategies between participants with mild and moderate IA levels. Independent sample t-tests were used to evaluate mean differences, and effect sizes were calculated to quantify the magnitude of these differences through Cohen’s *d*.

Pearson’s correlation coefficients were computed to examine the relationships among affective temperaments, emotion regulation strategies, and Internet addiction. These analyses were conducted to identify patterns of association and potential covariates for further modeling.

To investigate the complex interplay between individual dispositions and technology use, we hypothesized that IA severity would act as a moderator in the relationship between affective temperaments and emotion regulation strategies. This rationale is based on conceptualizing the problematic Internet use as an external tool for emotional regulation, which may alter the strength of the association between an individual’s temperament and their regulation strategies. Thus, moderation analyses were conducted to explore whether IA severity moderated the relationship between affective temperaments (predictors) and emotion regulation strategies (Reappraisal and Suppression) as dependent variables. Linear regression models included interaction terms to test moderation effects, with estimates, confidence intervals, and R-squared to evaluate the significance and strength of these interactions. Predictors were mean-centered to reduce multicollinearity, and 95% confidence intervals were estimated using bootstrapping (5,000 resamples) to ensure robust parameter estimation. Moreover, an *a priori* power analysis was conducted to determine a sufficient sample size for our moderation analyses. Using an alpha of 0.05, a desired power of 0.80, and a model with three predictors, the analysis indicated that a sample of 77 participants would be required to detect a medium effect size (Cohen’s *f^2^* = 0.15).

## Results

### Demographic and behavioral characteristics

The study sample comprised 262 participants, with 62.6% identifying as female (*N* = 164) and 37.4% as male (*N* = 98). The mean age of participants was 17.6 years (SD = 2.7; range: 13–21 years), and the mean years of education completed was 10.5 (SD = 2.9; range: 8–17 years). Most participants (93.1%) were students, with smaller proportions identifying as employees (4.2%), self-employed (1.9%), or unemployed (0.8%). The majority (89.7%) reported using smartphones as their primary device, and 71.4% reported engaging in daily online activities between 3 and 7 h (Table 1).

**Table 1:** Demographic characteristics of the study sample.

Variable		Value
Gender	Female	164 (62.6%)
	Male	98 (37.4%)
Age	Mean (SD)	17.6 (2.7)
	Range	13.0–21.0
Education (years)	Mean (SD)	10.25 (2.49)
	Range	8.0–17.0
Employment	Student	244 (93.1%)
	Employee	11 (4.2%)
	Self-employed	5 (1.9%)
	Unemployed	2 (0.8%)
Most used device	Computer	15 (5.7%)
	Tablet	12 (4.6%)
	Smartphone	235 (89.7%)
Daily online activity (hours)	Less than 3	32 (12.2%)
	From 3 to 7	187 (71.4%)
	More than 7	43 (16.4%)

### Group comparisons

In the group comparisons, participants with a moderate level of techno-dependence scored significantly higher on the Cyclothymic [*t*(260) = −3.173, *p* = 0.002, *d* = −0.396], Depressive [*t*(260) = −3.570, *p* < 0.001, *d* = −0.445], and Irritable [*t*(260) = −4.016, *p* < 0.001, *d* = −0.501] temperaments compared to those with a mild level of techno-dependence. No significant differences were observed for the Hyperthymic or Anxious temperaments.

In the Suppression dimension of the Emotion Regulation Questionnaire (ERQ), participants with a moderate IA severity had higher scores [*t*(260) = −2.617, *p* = 0.009, *d* = −0.3265] than those with mild. Conversely, in the Reappraisal dimension of the ERQ, participants with a mild IA severity scored significantly higher [*t*(260) = 2.477, *p* = 0.014, *d* = 0.3090] than those with a moderate level (Table 2).

**Table 2:** Characteristics of study variables in the two IA severity groups.

Variable		IA severity	Mean	SD	*t* (df)	*p*	*d*
Affective Temperaments	Cyclothymic	Mild	6.15	3.02	−3.173 (260)	**0.002**	**−0.396**
		Moderate	7.35	3.08			
	Depressive	Mild	2.87	1.98	−3.570 (260)	**<0.001**	**−0.445**
		Moderate	3.80	2.22			
	Irritable	Mild	1.76	1.74	−4.016 (260)	**<0.001**	**−0.501**
		Moderate	2.69	2.00			
	Hyperthymic	Mild	4.28	2.00	0.937 (260)	0.350	0.117
		Moderate	4.04	1.94			
	Anxious	Mild	1.67	1.12	0.176 (260)	0.860	0.022
		Moderate	1.65	1.16			
Emotion Regulation	Reappraisal	Mild	29.07	7.95	2.477 (260)	**0.014**	**0.309**
		Moderate	26.65	7.72			
	Suppression	Mild	15.68	4.83	−2.617 (260)	**0.009**	**−0.327**
		Moderate	17.32	5.23			

Value reported in bold are statistically significant at *p < 0.05.

### Correlations

Significant positive correlations were found between Internet addiction and Cyclothymic (*r* = 0.284, *p* < 0.001), Depressive (*r* = 0.329, *p* < 0.001), and Irritable (*r* = 0.357, *p* < 0.001) temperaments. Internet addiction was negatively correlated with Reappraisal (*r* = −0.144, *p* = 0.03) and positively correlated with Suppression dimensions (*r* = 0.185, *p* = 0.002). Furthermore, significantly negative correlations emerge between the Reappraisal dimension and Depressive (*r* = −0.129, *p* < 0.05) and Irritable (*r* = −0.250, *p* < 0.001) temperaments. Finally, the Suppression dimension correlates significantly with Cyclothymic (*r* = 0.191, *p* < 0.01) and Depressive (*r* = 0.269, *p* < 0.001) temperaments and negatively with Hyperthymic (*r* = −0.201, *p* < 0.01) temperament (Table 3).

**Table 3:** Correlation matrix of study variables.

Variable		1	2	3	4	5	6	7	8
1	Cyclothymic	—							
2	Depressive	0.484	—						
3	Irritable	0.425***	0.400***	—					
4	Hyperthymic	−0.051	−0.209***	0.051	—				
5	Anxious	0.287***	0.212***	0.125*	0.022	—			
6	Reappraisal	−0.041	−0.129*	−0.250***	0.095	0.056	—		
7	Suppression	0.191**	0.269***	0.053	−0.201**	0.047	0.020	—	
8	IA severity	0.284***	0.329***	0.357***	−0.119	0.023	−0.144*	0.185**	—

*p < 0.05, **p < 0.01, ***p < 0.001.

### Moderation analysis

A series of moderation analyses were conducted to examine whether IA severity moderated the relationships between affective temperaments (Depressive and Anxious) and emotion regulation strategies (Reappraisal and Suppression). Interaction terms were included in the models to assess the significance of these moderation effects.

**Figure 1:**
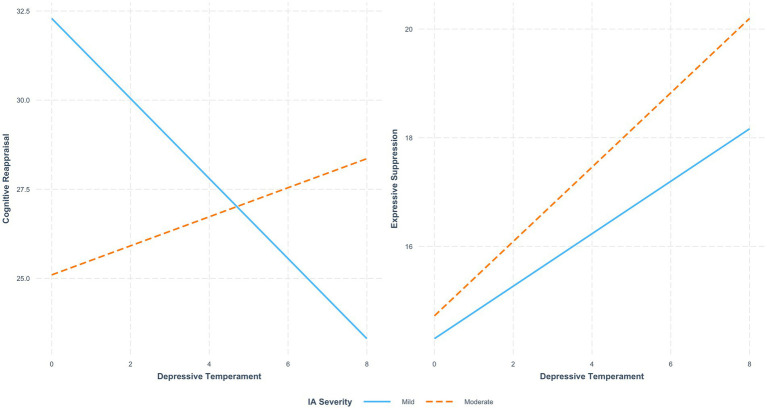
The moderating effect of Internet Addiction (IA) severity on the relationship between Depressive Temperament and **(A)** Reappraisal and **(B)** Suppression. The solid blue line represents Mild IA, and the dashed orange line represents Moderate IA.

Results indicated that Depressive Temperament was significantly associated with lower Reappraisal scores (*b* = −1.122, SE = 0.319, *t* = −3.523, *p* = 0.001), suggesting that individuals with higher depressive traits tend to engage less in cognitive reappraisal as an emotion regulation strategy. Similarly, IA severity was found to be a significant negative predictor of Reappraisal (*b* = −7.191, SE = 1.813, *t* = −3.967, *p* < 0.001). More importantly, a significant interaction effect was observed (*b* = 1.530, SE = 0.456, *t* = 3.355, *p* = 0.001), indicating that the negative association between Depressive Temperament and Reappraisal was weaker among individuals with moderate IA severity (Figure 1A). This moderation model was significant (*F* = 6.798, *p* < 0.05) and explained 7.3% of the variance in Reappraisal (*R*^2^ = 0.073).

For Suppression, results showed that Depressive Temperament was significantly associated with higher Suppression scores (*b* = 0.483, SE = 0.202, *t* = 2.391, *p* = 0.018), indicating that individuals with depressive traits are more likely to rely on expressive suppression as an emotion regulation strategy. However, neither IA severity (*b* = 0.420, SE = 1.149, *t* = 0.365, *p* = 0.715) nor the interaction between Depressive Temperament and IA severity (*b* = 0.201, SE = 0.289, *t* = 0.697, *p* = 0.486) significantly predicted Suppression (Figure 1B), suggesting that IA severity does not moderate this relationship (*F* = 7.967, *R*^2^ = 0.085) (Table 4).

**Table 4:** Moderation analysis of depressive temperament (DT) on reappraisal and suppression with IA severity as moderator.

ERQ dimension	Predictor	Estimate	SE	*t*	*p*	LLCI	ULCI	*F*	*R* ^2^
Reappraisal	*Intercept*	32.291	1.109	29.122	0.000	30.107	34.474	6.798	0.073
	DT	−1.122	0.319	−3.523	**0.001**	−1.750	−0.495		
	IA	−7.191	1.813	−3.967	**0.000**	−10.761	−3.622.		
	DT * IA	1.530	0.456	3.355	**0.001**	0.632	2.428		
Suppression	*Intercept*	14.301	0.703	20.344	0.000	12.917	15.685	7.967	0.085
	DT	0.483	0.202	2.391	**0.018**	0.085	0.881		
	IA	0.420	1.149	0.365	0.715	−1.843	2.683		
	DT * IA	0.201	0.289	0.697	0.486	−0.368	0.771		

Value reported in bold are statistically significant at *p < 0.05.

**Figure 2:**
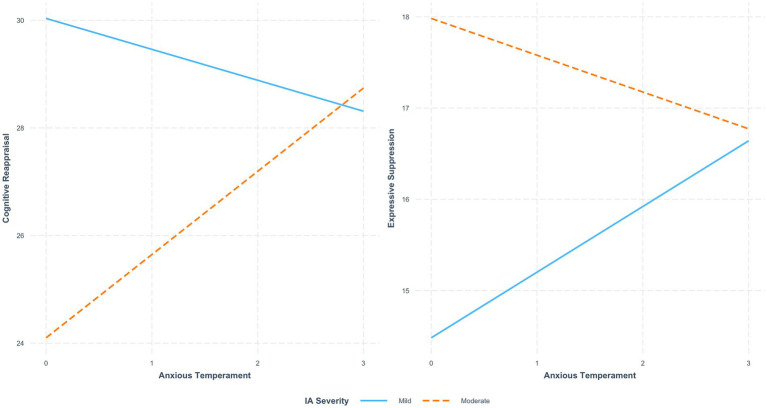
The moderating effect of Internet Addiction (IA) Severity on the relationship between Anxious Temperament and **(A)** Reappraisal and **(B)** Suppression. The solid blue line represents Mild IA, and the dashed orange line represents Moderate IA.

Regarding Anxious Temperament, no significant direct effect on Reappraisal was found (*b* = −0.575, SE = 0.569, *t* = −1.009, *p* = 0.314), but IA severity significantly predicted lower Reappraisal scores (*b* = −5.934, SE = 1.711, *t* = −3.468, *p* < 0.001). A significant interaction effect emerged (*b* = 2.121, SE = 0.850, *t* = 2.495, *p* = 0.013), suggesting that the negative association between Anxious Temperament and Reappraisal was weaker among individuals with moderate IA severity (Figure 2A). The overall model was significant (*F* = 4.425, *p* < 0.05) and accounted for 4.9% of the variance in Reappraisal (*R*^2^ = 0.049).

For Suppression, Anxious Temperament was significantly associated with higher Suppression scores (*b* = 0.720, SE = 0.364, *t* = 1.977, *p* = 0.049), and IA severity was also a significant positive predictor (*b* = 3.501, SE = 1.094, *t* = 3.199, *p* = 0.002). The interaction effect was significant (*b* = −1.123, SE = 0.544, *t* = −2.066, *p* = 0.040), indicating that the positive association between Anxious Temperament and Suppression was weaker among individuals with moderate IA severity (Figure 2B). The full model was significant (*F* = 3.945, *p* < 0.05), explaining 4.4% of the variance in Suppression (*R*^2^ = 0.044) (Table 5).

**Table 5:** Moderation analysis of anxious temperament (AT) on reappraisal and suppression with IA severity as moderator.

ERQ dimension	Predictor	Estimate	SE	*t*	*p*	LLCI	ULCI	*F*	*R* ^2^
Reappraisal	*Intercept*	30.034	1.145	26.229	<0.001	27.779	32.289	4.425	0.049
	AT	−0.575	0.569	−1.009	0.314	−1.695	0.546		
	IA	−5.934	1.711	−3.468	**<0.001**	−9.303	−2.565		
	AT * IA	2.121	0.850	2.495	**0.0132**	0.447	3.795		
Suppression	*Intercept*	14.481	0.732	19.772	<0.001	13.039	15.924	3.945	0.044
	AT	0.720	0.364	1.977	**0.049**	0.003	1.437		
	IA	3.501	1.094	3.199	**0.002**	1.346	5.656		
	AT * IA	−1.123	0.544	−2.066	**0.040**	−2.194	−0.053		

Value reported in bold are statistically significant at *p < 0.05.

## Discussion

This study explored the moderating role of IA severity on the relationship between affective temperaments and emotion regulation strategies in adolescents and young adults with Internet addiction. The results reveal that affective temperaments, particularly cyclothymic, depressive and irritable, are closely related to problematic Internet use and specific emotion regulation difficulties, offering important insights related to temperamental predispositions and subsequent adaptive and maladaptive coping strategies enacted in digital contexts.

Consistent with previous research (Ozturk et al., 2013; Mento et al., 2024), our results confirm that affective temperaments have relationships with problematic Internet use. This was first evident in the group comparison analysis, where participants with moderate IA reported significantly higher levels of cyclothymic, depressive, and irritable temperaments than the mild IA group.

Reinforcing this finding, the broader correlational analysis showed that these same temperaments showed significant positive associations with IA suggesting that individuals with greater emotional instability, negative affectivity, and irritability may be more vulnerable to problematic Internet use. These results align with prior studies indicating that mood instability and emotional dysregulation are key predictors of compulsive Internet use (Rahme et al., 2022; Jović et al., 2024). The lack of significant associations with hyperthymic temperament suggests that emotional stability may act as protective factors against excessive Internet use.

Our results also highlight important links between affective temperaments and specific emotion regulation strategies. Depressive and irritable temperaments were significantly associated with lower cognitive reappraisal, indicating that individuals with greater emotional distress and instability have difficulty restructuring their negative emotions. This finding aligns with evidence suggesting that depressive traits are linked to rigid, maladaptive cognitive styles that impair effective reappraisal (Troy et al., 2010).

Suppression was positively associated with cyclothymic and depressive temperaments, suggesting that individuals with mood instability and negative affectivity tend to rely on suppression rather than engaging in active emotional processing.

These findings are consistent with prior studies showing that individuals with depressive traits are more prone to emotional inhibition as a coping mechanism (Joormann and Gotlib, 2010). Suppression was also negatively associated with hyperthymic temperament, reinforcing the role of positive affectivity and emotional stability in reducing reliance on suppression. However, the literature on the relationship between affective temperaments and emotion regulation strategies remains limited, highlighting the need for further research to clarify this association and explore potential moderating variables. In line with previous studies (Amendola et al., 2019; Yang et al., 2022), our results suggest that difficulties in emotion regulation play a crucial role in IA. Specifically, individuals with moderate IA severity exhibit greater reliance on suppression and reduced use of cognitive reappraisal, highlighting a maladaptive emotion regulation pattern.

Our analyses show that depressive temperament is negatively associated with reappraisal, meaning individuals with higher depressive traits struggle with cognitive restructuring. Moreover, IA severity appears to moderate this effect, as individuals with moderate IA exhibit a weaker negative association, or even a slight positive association, between depressive temperament and reappraisal.

Nevertheless, this moderation effect does not imply that IA fully compensates for difficulties in reappraisal. While digital tools may offer external cognitive support, their long-term impact on autonomous emotion regulation skills remains uncertain. Structured online interactions may provide temporary benefits, but excessive reliance on digital resources could hinder the development of independent cognitive restructuring abilities. Therefore, problematic Internet use should be considered a complementary rather than a substitutive mechanism for emotion regulation (Liang et al., 2021).

Unexpectedly, while IA severity moderates the association between depressive temperament and reappraisal, it does not influence suppression. This suggests that emotional inhibition in depressive individuals is a stable trait, independent of technology use. Although problematic Internet use has been considered a compensatory mechanism for emotion regulation difficulties (Gioia et al., 2021), our findings indicate that it does not necessarily reinforce suppression, which appears to persist across both digital and offline contexts.

Finally, the data suggests that anxious temperament is not directly associated with cognitive reappraisal, suggesting that this temperamental dimension may not significantly influence reappraisal strategies. However, anxious temperament is linked to a greater tendency toward emotional suppression, indicating that individuals with this predisposition tend to inhibit emotional expression, likely as an avoidance strategy (Llewellyn et al., 2013). Moreover, IA severity moderates both associations. The negative association between anxious temperament and cognitive reappraisal is weaker in individuals with moderate IA, suggesting that technology use may provide cognitive support for emotional reprocessing. Simultaneously, the positive association between anxious temperament and suppression is less pronounced in individuals with moderate IA, which could indicate that digital interactions offer alternative spaces for emotional expression. However, these effects do not exclude the risk that excessive reliance on technology may reinforce emotional avoidance mechanisms in the long term.

These moderation effects can be also interpreted through the Interaction of Person-Affect-Cognition-Execution (I-PACE) model for addictive behaviors (Brand et al., 2016, 2019). The model posits that predisposing variables (the ‘Person’ component), such as the affective temperaments identified in our study, contribute to specific affective and cognitive responses to external triggers. In this context, the internet provides a platform for maladaptive coping (the ‘Execution’ component). Our finding that IA severity moderates the temperament-regulation link supports this framework; individuals with depressive or anxious traits may use the internet as a readily available coping mechanism to manage negative affect, which manifests as a form of cognitive scaffolding. While this may offer short-term relief, the I-PACE model suggests this reinforces a dysfunctional cycle, potentially hindering the development of autonomous, internal emotion regulation skills over the long term (Brand et al., 2025; Chen et al., 2025).

The expansion of digital technologies in mental health provides new opportunities for adaptive emotion regulation. Social media and online platforms enable real-time emotional assessment and cognitive restructuring through supportive content and interactive communities (Bettis et al., 2022; Scott et al., 2024). However, prolonged reliance on these tools may foster emotional disengagement, reinforce suppression and reducing face-to-face emotional expression over time (Baker et al., 2016).

Our findings may reflect a specific characteristic of population, who have grown up with continuous access to connected technologies. In this population, digital tools may act as cognitive and emotional supports, helping with emotion regulation and self-monitoring during a developmental phase marked by identity formation. While this support can offer temporary benefits, it may also influence how emotional and social skills develop.

This interpretation is consistent with research showing that adaptive scaffolding enhances learning and emotional engagement in digital environments (Faber et al., 2024; Chen and Hou, 2025). Similarly, the constant presence of smartphones may function as a personalized aid for regulating emotions — especially for individuals with vulnerable temperaments.

Future research should examine whether similar scaffolding patterns are present in non-digital natives, and whether different forms of digital engagement led to more adaptive or maladaptive emotional outcomes. Exploring these mechanisms across generations and behaviors could help design interventions that make good use of technology without reinforcing emotional avoidance.

Moreover, evidence suggests that neurobiological alterations observed in individuals with Internet addiction reflect vulnerability factors rather than deterministic causes, underscoring the role of psychological and environmental influences in the development of IAD (Brand et al., 2016, 2019, 2025).

Problematic Internet use, in this perspective, may not only represent a pathological risk but also a way of thinking and coping shaped by cultural habits, especially among digital natives.

## Conclusion

This study highlights the central role of affective temperaments in emotion regulation and their connection to IA in adolescents and young adults with problematic Internet use. The results suggest that affective temperaments characterized by emotional instability and negative affectivity are more vulnerable to IA and tend to adopt less adaptive emotion regulation strategies, such as emotional suppression.

Moreover, problematic Internet use emerges as a complex moderating factor: while it may mitigate the negative effects of depressive temperament on cognitive reappraisal, it also appears to reinforce the use of emotional suppression, potentially contributing to emotional disengagement and a reduction in face-to-face interactions.

This suggests that moderate IA severity might act as a “buffer,” potentially providing external cognitive scaffolding through technology-mediated coping mechanisms, such as engaging with online content that facilitates reappraisal.

These findings underscore the importance of a balanced approach to technology use, for promotion of a conscious and critical use of technology through digital education, cognitive enhancement and emotional regulation, starting in the school context. Nevertheless, the long-term impact of IA remains an open question, as prolonged and dysfunctional technology use may still contribute to emotional disengagement and reduced face-to-face interactions.

## Limitations

Our results should be interpreted with caution because of several limitations, particularly the small sample size consisting mainly of women and the cross-sectional design, which may have reduced the overall impact of the study. In addition, the use of the IAT-20 scale did not discriminate specific forms of Internet activity, such as gaming, social-networking, and binge-watching. Although the moderation analyses yielded statistically significant interaction effects, the explained variance (R^2^ values ranging from 4.4 to 8.5%) suggests that other unmeasured factors likely contribute to the relationship between affective temperaments, emotion regulation, and IA severity. This indicates that while temperamental traits play a role in problematic Internet use, additional psychological and environmental variables—such as impulsivity, peer influence, or underlying psychopathology—should be considered in future research to develop a more comprehensive explanatory model.

## Data Availability

The raw data supporting the conclusions of this article will be made available by the authors, without undue reservation.
